# Dehydrocorydaline Accelerates Cell Proliferation and Extracellular Matrix Synthesis of TNFα-Treated Human Chondrocytes by Targeting Cox2 through JAK1-STAT3 Signaling Pathway

**DOI:** 10.3390/ijms23137268

**Published:** 2022-06-30

**Authors:** Yongqiang Sha, Beibei Zhang, Liping Chen, Chunli Wang, Tao Sun

**Affiliations:** 1 Center for Precision Medicine, School of Medicine and School of Biomedical Sciences, Huaqiao University, Xiamen 361021, China; zhangbeibei2299@163.com (B.Z.); ch990721@163.com (L.C.); 2 National Innovation and Attracting Talent “111” Base, Key Laboratory of Biorheological Science and Technology, Ministry of Education, College of Bioengineering, Chongqing University, Chongqing 400030, China; lilywang@cqu.edu.cn

**Keywords:** dehydrocorydaline, osteoarthritis, cell proliferation, extracellular matrix, chondrocytes

## Abstract

Osteoarthritis (OA) causes severe degeneration of the meniscus and cartilage layer in the knee and endangers joint integrity and function. In this study, we utilized tumor necrosis factor α (TNFα) to establish in vitro OA models and analyzed the effects of dehydrocorydaline (DHC) on cell proliferation and extracellular matrix (ECM) synthesis in human chondrocytes with TNFα treatment. We found that TNFα treatment significantly reduced cell proliferation and mRNA and protein expression levels of aggrecan and type II collagen, but caused an increase in mRNA and protein expression levels of type I collagen, matrix metalloproteinase 1/13 (MMP1/13), and prostaglandin-endoperoxide synthase 2 (PTGS2, also known as Cox2) in human chondrocytes. DHC significantly promoted the cell activity of normal human chondrocytes without showing cytotoxity. Moreover, 10 and 20 μM DHC clearly restored cell proliferation, inhibited mRNA and protein expression levels of type I collagen, MMP 1/13, and Cox2, and further increased those of aggrecan and type II collagen in the TNFα-treated human chondrocytes. RNA transcriptome sequencing indicated that DHC could improve TNFα-induced metabolic abnormalities and inflammation reactions and inhibit the expression of TNFα-induced inflammatory factors. Furthermore, we found that the JAK1-STAT3 signaling pathway was confirmed to be involved in the regulatory effects of DHC on cell proliferation and ECM metabolism of the TNFα-treated human chondrocytes. Lastly, to explore the effects of DHC in vivo, we established an anterior cruciate ligament transection (ACLT)-stimulated rat OA model and found that DHC administration significantly attenuated OA development, inhibited the enzymatic hydrolysis of ECM, and reduced phosphorylated JAK1 and STAT3 protein expression in vivo after ACLT for 6 weeks. These results suggest that DHC can effectively relieve OA progression, and it has a potential to be utilized for the clinical prevention and therapy of OA as a natural small molecular drug.

## 1. Introduction

Cartilage in the knee joint has a smooth surface and soft matrix stiffness and is covered by viscous joint fluid. The cartilage can therefore buffer the mechanical stress between the tibia and femur during exercise and decrease friction [1]. Aging and aberrant mechanical load-caused anterior cruciate ligament (ACL) rupture will lead to the cell loss of chondrocytes and extracellular matrix (ECM) degradation in articular cartilage and further cause osteoarthritis (OA) [2,3]. Once OA occurs, inflammatory factors are highly expressed and pervasive in the synovial fluid, and then the matrix metalloproteinases (MMPs) is activated, further adding to the degradation of ECM [4,5]. All these changes affect the mechanical distribution from femur to tibia, accelerating OA progression [6]. As reported previously, abnormal mechanical environmental conditions, the limitation of cell proliferation, and a reduction in the ECM synthesis of the chondrocytes make it too difficult for the articular cartilage to heal on its own [7,8,9].

OA is a complex pathological process in chronic inflammatory diseases [10]. The amount of inflammatory factors and chronic inflammation are the critical issues leading to the continuous deterioration caused by OA, reducing cell proliferative capacity, inducing cell loss or apoptosis, increasing matrix metalloproteinase (MMPs) expression, and endangering the ECM synthesis in chondrocytes [11,12]. Therefore, anti-inflammatory therapies always show positive effects in OA treatment. A number of growth factors, cytokines, and small molecules drugs have been confirmed to be effective for attenuating OA progression by resisting inflammatory reaction in the tumor necrosis factor α (TNFα)- or proinflammatory interleukin (IL)-treated chondrocytes, such as safflower yellow [13], transforming growth factor β (TGFβ) [14], lysyl oxidase [15], and so on. Small molecules drugs have attracted more interest nowadays.

Dehydrocorydoline (DHC), as a natural small molecules drug, has been confirmed to be the major bioactive component of *Corydalis yanhusuo* and has a variety of regulatory effects [16]. DHC shows excellent effects on anti-tumor activity, such as in non-small lung cancer metastasis or melanoma development, and even reduces bone cancer pain effectively [17,18,19]. In addition, DHC can ameliorate depressive symptoms via inhibiting uptake-2 monoamine transporters and avoid neural damage by inhibiting glutamate release [20,21]. Importantly, DHC exhibits excellent anti-inflammatory effects and significantly reduces TNFα, IL-1β, and IL-6 expression to alleviate inflammatory pain or cardiovascular diseases [22,23]. Nevertheless, DHC’s benefit for attenuating OA development is still unknown and needs to be explored in detail.

In the current study, TNFα was applied in human chondrocytes to simulate inflammatory environmental conditions and OA in vitro. Subsequently, DHC was utilized to analyze its effects on cell proliferation, ECM synthesis, and ECM-related degrading enzyme expression levels in TNFα-treated human chondrocytes after detecting the cytotoxicity of DHC on human chondrocytes. In addition, transcriptome sequencing was performed to screen the underlying signaling pathways involved in DHC’s regulation of the abovementioned indexes and the expression levels of inflammatory factors in TNFα-treated human chondrocytes. Furthermore, rat OA models were established by carrying out complete ACL transection (ACLT) surgery. DHC administration was applied in the following 6 weeks according to the experimental design, and DHC’s ability to facilitate OA treatment in vivo and attenuate ECM degradation in the articular cartilage was also analyzed. Potential molecular mechanisms were simply verified simultaneously.

## 2. Results

### 2.1. DHC Facilitated Cell Proliferation of the TNFα-Treated Human Chondrocytes

**Figure 1:**
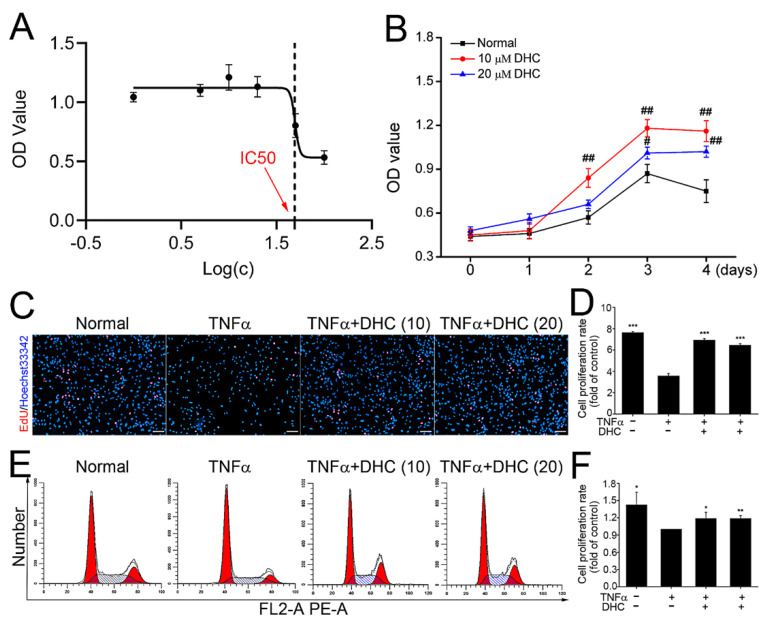
Dehydrocorydaline (DHC) ameliorated cell proliferative capacity of the TNFα-treated human chondrocytes. (**A**) 50% inhibiting concentration (IC50) of DHC on the normal human chondrocytes after culture for 48 h. (**B**) Effects of 10 and 20 μM DHC on cell activities of normal human chondrocytes after culture for 1, 2, 3, and 4 days. (**C**) EdU-positive cells in the normal, TNFα, TNFα + 10 μM DHC, and TNFα + 20 μM DHC groups and (**D**) the quantitative results after treatment for 48 h. (**E**) Cell proliferation of the human chondrocytes in the normal, TNFα, TNFα + 10 μM DHC, and TNFα + 20 μM DHC groups detected by flow cytometry analysis and (**F**) the quantitative results after treatment for 48 h. Scar bar = 100 μm. Data are presented as mean ± SD. *, *p* < 0.05; **, *p* < 0.01; ***, *p* < 0.001 compared to the control group (the TNFα group). #, *p* < 0.05; ##, *p* < 0.01 compared to the normal group at the same time point.

The cytotoxicity of DHC was characterized by 50% inhibiting concentration (IC50), and the results indicated that the IC50 of DHC on human chondrocytes was 49.65 μM (Figure 1A). Subsequently, 10 and 20 μM DHC were further applied in human chondrocytes for 4 days, and cell activities were verified each day. After treatment at 2, 3, and 4 days, 10 μM DHC significantly increased the cell activity of human chondrocytes from 0.57 ± 0.04 to 0.84 ± 0.06 (*p* < 0.01), from 0.87 ± 0.06 to 1.18 ± 0.06 (*p* < 0.01), and from 0.75 ± 0.08 to 1.16 ± 0.07 (*p* < 0.01), respectively, compared with normal human chondrocytes (Figure 1B). However, 20 μM DHC only remarkably raised the cell activity of human chondrocytes to 1.01 ± 0.04 (*p* < 0.05) at day 3 and 1.02 ± 0.04 (*p* < 0.01) at day 4, showing a slightly worse pro-proliferative effect (Figure 1B). Furthermore, the effects of DHC on the cell proliferative rates of TNFα-treated human chondrocytes were detected through EdU staining and flow cytometry at day 2. As shown in Figure 1C, TNFα clearly reduced the EdU-positive cell rate from 7.64 ± 0.10% to 3.57 ± 0.22% (*p* < 0.001). Compared with the TNFα group, 10 and 20 mM DHC increased the proliferative rates of the TNFα-treated human chondrocytes to 6.93 ± 0.15% (*p* < 0.001) and 6.45 ± 0.14% (*p* < 0.001), respectively (Figure 1C,D). For flow cytometry analysis, cells in the S + G2/M phases were counted as the proliferating cells. It was found that TNFα could markedly inhibit the cell proliferation of human chondrocytes, whereas 10 and 20 μM DHC restored the proliferative capacity of the TNFα-treated human chondrocytes (Figure 1E,F).

### 2.2. DHC Improved ECM Synthesis and Degradation in the TNFα-Treated Human Chondrocytes

**Figure 2:**
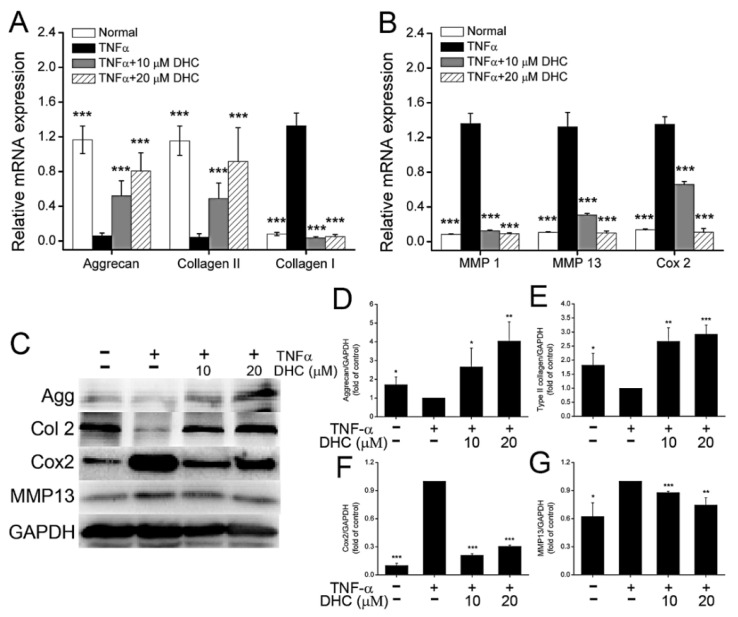
Dehydrocorydaline (DHC) improved the mRNA and protein expression levels of extracellular matrix synthesis (ECM)-relevant genes in the TNFα-treated human chondrocytes at 48 h. Effects of DHC on the mRNA expression levels of (**A**) aggrecan (Agg), type I/II collagen, and (**B**) degrading enzymes such as matrix metalloproteinase 1/13 (MMP1/13) and prostaglandin-endoperoxide synthase 2 (PTGS2, also known as Cox2) in the TNFα-treated human chondrocytes. (**C**) Protein expression levels of Agg, type II collagen, MMP13, and Cox2 in the TNFα-treated human chondrocytes detected through western blotting and (**D**–**G**) the quantitative results of the bands. Data are presented as mean ± SD. *, *p* < 0.05; **, *p* < 0.01; ***, *p* < 0.001 compared to the TNFα group.

After culture for 48 h, the effects of DHC on the mRNA expression levels of aggrecan, type I/II collagen, MMP1/13, and prostaglandin-endoperoxide synthase 2 (PTGS2, also known as Cox2) in the TNFα-treated human chondrocytes were analyzed separately. The findings showed that TNFα significantly reduced the mRNA expression levels of aggrecan and type II collagen from 1.17 ± 0.16 to 0.06 ± 0.03 (*p* < 0.001), and from 1.16 ± 0.17 to 0.05 ± 0.04 (*p* < 0.001), whereas it increased the mRNA expression of type I collagen, MMP1, MMP13 and Cox2 from 0.08 ± 0.02 to 1.33 ± 0.15 (*p* < 0.001), from 0.08 ± 0.01 to 1.36 ± 0.12 (*p* < 0.001), from 0.11 ± 0.01 to 1.33 ± 0.17 (*p* < 0.001), and from 0.14 ± 0.01 to 1.35 ± 0.09 (*p* < 0.001) in the TNFα-treated human chondrocytes, respectively (Figure 2A,B). DHC exhibited excellent regulatory effects on ECM synthesis in the TNFα-treated human chondrocytes. Compared with the TNFα group, 10 and 20 μM DHC could obviously increase the mRNA expression levels of aggrecan to 0.52 ± 0.17 (*p* < 0.001) and 0.81 ± 0.21 (*p* < 0.001) and the type II collagen to 0.49 ± 0.18 (*p* < 0.001) and 0.92 ± 0.39 (*p* < 0.001), but it decreased the mRNA expression levels of type I collagen to 0.04 ± 0.01 (*p* < 0.001) and 0.05 ± 0.02 (*p* < 0.001), and MMP1 to 0.13 ± 0.01 (*p* < 0.001) and 0.09 ± 0.01 (*p* < 0.001), and MMP13 to 0.31 ± 0.02 (*p* < 0.001) and 0.10 ± 0.02 (*p* < 0.001), and Cox2 to 0.66 ± 0.03 (*p* < 0.001) and 0.11 ± 0.04 (*p* < 0.001) in the TNFα-treated human chondrocytes, respectively (Figure 2A,B).

Protein expression levels of aggrecan, type II collagen, Cox2, and MMP13 in human chondrocytes in the normal, TNFα, TNFα + 10 μM DHC, and TNFα + 20 μM DHC groups were furtherly detected. The results exhibited a similar trend with that of the mRNA expression. TNFα significantly inhibited the expression levels of aggrecan and type II collagen, whereas it promoted the expression levels of Cox2 and MMP13. Nevertheless, DHC treatment could effectively impair the adverse effects of TNFα on human chondrocytes (Figure 2C–G).

### 2.3. Detection of Differently Expressed Genes, GO, and KEGG Enrichment Analysis

**Figure 3:**
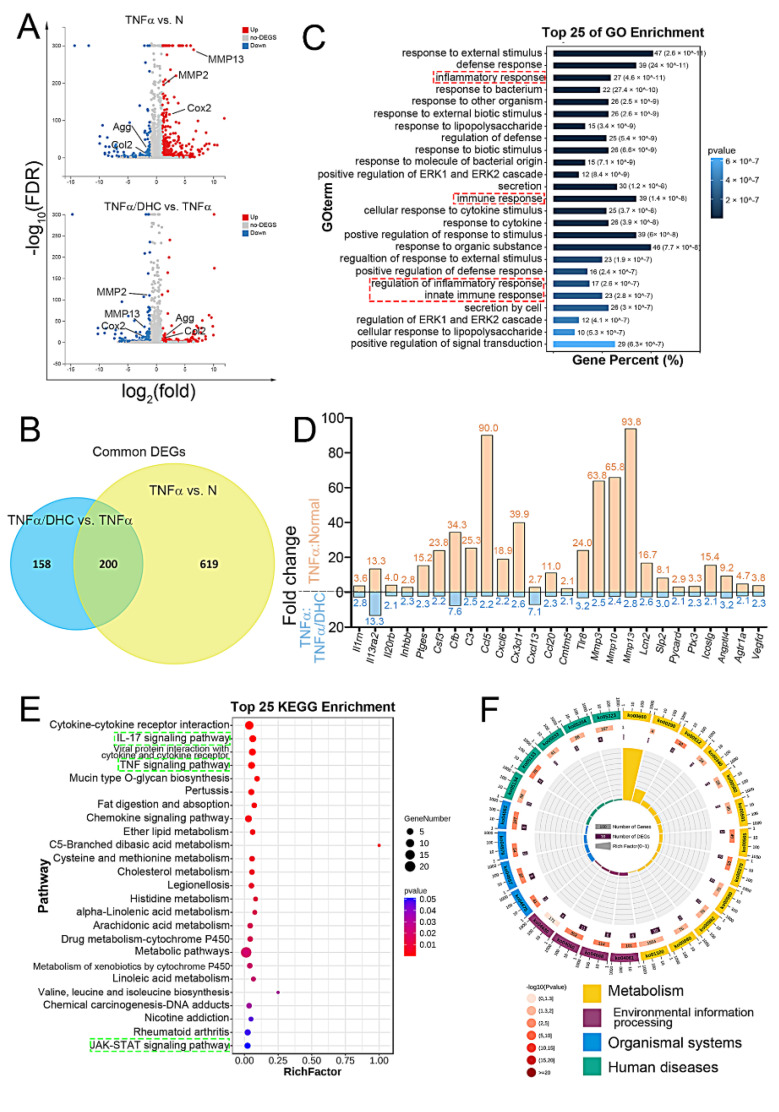
Dehydrocorydaline (DHC) regulated the gene expression profiles in the TNFα-treated human chondrocytes after 48 h. (**A**) Differently expressed genes (DEGs) in human chondrocytes between the normal group and TNFα group (819 DEGs), and TNFα group and TNFα + 10 μM DHC group (358 DEGs), were analyzed through Volcano plot. (**B**) Common DEGs of the 819 and 358 DEGs detected through Venn diagram analysis. (**C**) Gene ontology (GO) analysis of the common DEGs. (**D**) Fold change (FC) expression of inflammatory factors. Kyoto Encyclopedia of Genes and Genomes (KEGG) analysis showed in (**E**) gradient and (**F**) circular diagram.

In consideration of 20 μM DHC’s slightly worse cytotoxicity and pro-proliferative effects, RNA sequencing was performed to analyze only the gene expression profiles in the human chondrocytes in the normal, TNFα, and TNFα + 10 μM DHC groups. The differently expressed genes (DEGs) in the human chondrocytes between the normal group and TNFα group, and the TNFα group and the TNFα + 10 μM DHC group, were analyzed through a Volcano plot. The genes with FDR (−log10) > 3 and log2(FC) > 1 were considered to be significant. As shown in Figure 3A, 819 DEGs exited between the normal and TNFα groups, e.g., aggrecan, type II collagen, Cox2 and MMP13, whereas 358 genes exited between the TNFα and TNFα + 10 μM DHC groups. Subsequently, a Venn diagram was constructed to analyze the commonly expressed genes of the 819 and 358 DEGs, and, finally, 200 genes were obtained (Figure 3B). The gene ontology (GO) analysis showed that the biological process of the common genes was enriched in the inflammatory response, the regulation of the inflammatory response, the immune response, substance metabolism, and the cell response to the stimulus (Figure 3C). The inflammatory factors in the common DEGs were further screened, and the fold change expression was counted. The results indicated that DHC obviously inhibited the TNFα-induced high expression of inflammatory factors (*p* < 0.001) (Figure 3D). Furthermore, an analysis in the Kyoto Encyclopedia of Genes and Genomes (KEGG) found that DEGs were majorly involved in regulating metabolism, environmental information processing, organism systems, and human diseases. The relevant signaling pathways were also enriched in cell metabolism and inflammation-relevant pathways, e.g., the IL-17 signaling pathway, the TNF signaling pathway, and the Jak-STAT signaling pathway (Figure 3E,F).

### 2.4. DHC Inhibited the Phosphorylated Jak1 and Stat3 Expression in the TNFα-Treated Human Chondrocytes

**Figure 4:**
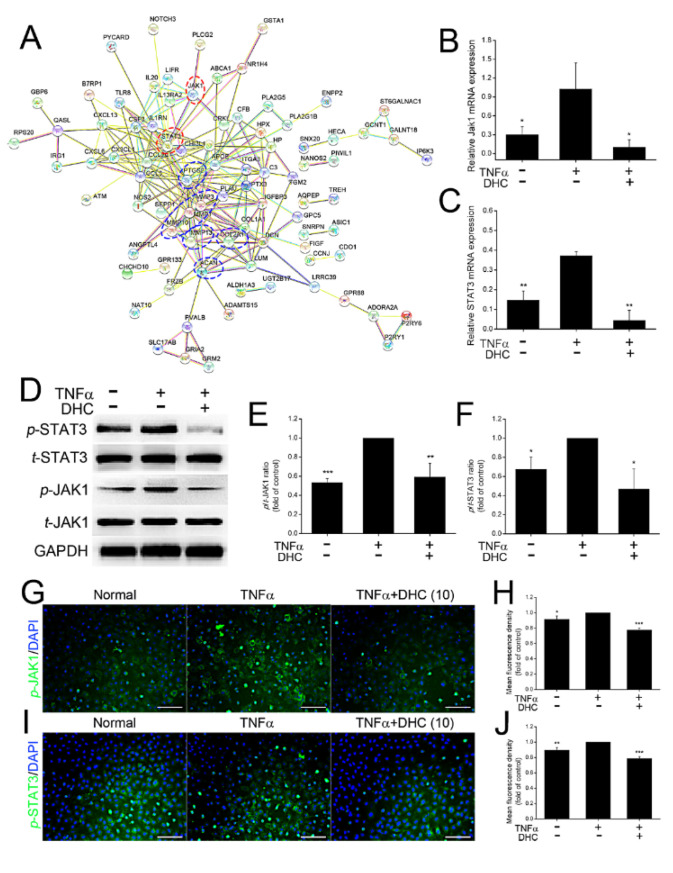
Dehydrocorydaline (DHC) inhibited the mRNA and phosphorylated JAK1 and STAT3 expression in the TNFα-treated human chondrocytes. (**A**) Protein interaction network analysis of the common differently expressed genes (DEGs). (**B**) JAK1 and (**C**) STAT3 mRNA expression levels in the human chondrocytes in the normal, TNFα and TNFα + 10 μM DHC groups. (**D**) Western blotting analysis and the quantified results of phosphorylated/total (*p*/*t*) ratio of (**E**) JAK1 and (**F**) STST3. (**G**) Immunofluorescence staining of *p*-JAK1 and (**H**) quantified results. (**I**) Immunofluorescence staining of p-STAT3 and (**J**) quantified results. Scale bar = 100 μm. Data are presented as mean ± SD. *, *p* < 0.05; **, *p* < 0.01; ***, *p* < 0.001 compared to the TNFα group.

The JAK-STAT signaling pathway is a critical underlying mechanism promoting inflammation and osteoarthritis, as reported previously [24,25]. In addition to the KEGG analysis, the protein interaction network analysis also found that JAK1-STAT3 might be the major signaling pathway for regulating the type I/II collagen, aggrecan, MMP1/13, and Cox2 expression (Figure 4A). Subsequently, mRNA and protein expression levels of JAK1 and STAT3 were detected. To stay consistent with RNA sequencing analysis, only the underlying mechanisms of 10 mM DHC-regulating cell proliferation and ECM synthesis of TNFα-treated human chondrocytes was further detected. Compared with the normal group, TNFα treatment increased the mRNA expression levels of JAK1 and STAT3 from 0.30 ± 0.13 to 1.03 ± 0.41 (*p* < 0.05) and from 0.15 ± 0.05 to 0.37 ± 0.02 (*p* < 0.01), whereas DHC administration could reduce their expression to 0.10 ± 0.12 (*p* < 0.05) and 0.04 ± 0.05 (*p* < 0.01) (Figure 4B,C). Protein expression levels of total and phosphorylated (*t* and *p*) JAK1 and STAT3 were also detected, and the ratio results of *p*/*t*-JAK1 and STAT3 were counted. The data found that the ratios of *p*/*t*-JAK1 and STAT3 were increased by 88.04% and 48.28% by TNFα, respectively, whereas DHC weakened the effects of TNFα and decreased the ratios of *p*/*t*-JAK1 and STAT3 to 59.28% and 46.78% (Figure 4D–F). Furthermore, the protein expression of p-JAK1 and p-STAT3 was also detected by immunofluorescence staining, and TNFα-induced high expression of *p*-JAK1, and p-STAT3 could also be reduced by DHC treatment (Figure 4G–J).

### 2.5. DHC Ameliorated Cell Proliferation and EXM Synthesis in the TNFα-Treated Human Chondrocytes via JAK1-STAT3 Pathway

**Figure 5:**
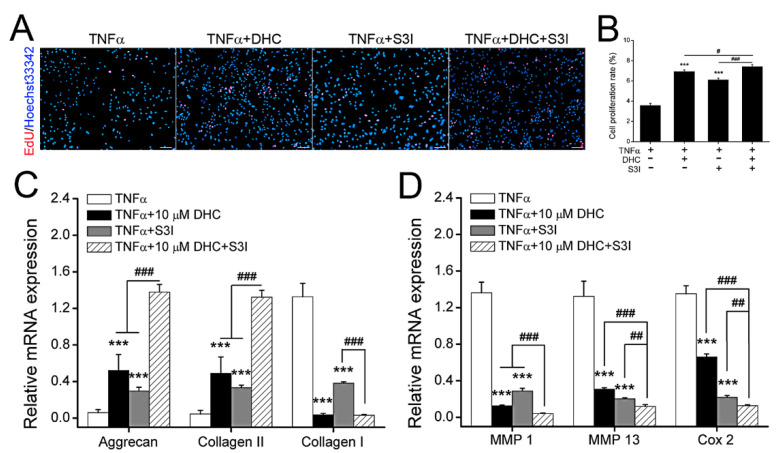
Dehydrocorydaline (DHC) regulated cell proliferation and extracellular matrix (ECM) synthesis in the TNFα-treated human chondrocytes via JAK1-STAT3 pathway. (**A**) EdU staining for detecting cell proliferation and (**B**) quantified results. The mRNA expression levels of (**C**) aggrecan (Agg), type I/II collagen, and (**D**) degrading enzymes such as matrix metalloproteinase 1/13 (MMP1/13) and prostaglandin-endoperoxide synthase 2 (PTGS2, also known as Cox2) in the TNFα-treated human chondrocytes. Scale bar = 100 μm. Data are presented as mean ± SD. ***, *p* < 0.001 compared to the TNFα group. #, *p* < 0.05; ##, *p* < 0.01, ###, *p* < 0.001 compared to the TNFα + DHC + S3I-201 (S3I) group.

The potential signaling pathway involved in DHC mediating cell proliferation and ECM synthesis and degradation was verified through EdU staining and RT-qPCR. Compared with the TNFα group, S3I-201 (S3I, inhibitor of JAK1-STAT3 pathway) increased the EdU positive cells from 3.57 ± 0.22% to 6.11 ± 0.16% (*p* < 0.001) (Figure 5A,B). Moreover, S3I could weaken the protective effects of DHC against TNFα, and the EdU positive cell ratio in the TNFα + S3I + DHC group was raised from 6.93 ± 0.15% to 7.42 ± 0.19% (*p* < 0.05) compared to the TNFα + DHC group (Figure 5A,B). Aggrecan, type II collagen, type I collagen, MMP1/13, and Cox2 mRNA expression levels in the TNFα, TNFα + DHC, TNFα + S3I, and TNFα + DHC +S3I groups were further detected. Compared with the TNFα group, S3I increased aggrecan and type II collagen mRNA expression from 0.06 ± 0.03 to 0.30 ± 0.04 (*p* < 0.001) and from 0.05 ± 0.04 to 0.33 ± 0.03 (*p* < 0.001), whereas it reduced type I collagen, MMP1, MM13, and Cox2 mRNA expression from 1.33 ± 0.15 to 0.38 ± 0.02 (*p* < 0.001), from 1.36 ± 0.12 to 0.29 ± 0.03 (*p* < 0.001), from 1.33 ± 0.17 to 0.20 ± 0.01 (*p* < 0.001), and from 1.35 ± 0.09 to 0.22 ± 0.02 (*p* < 0.001), respectively (Figure 5C,D). In addition, compared with the TNFα + DHC group, S3I also increased the aggrecan and type II collagen mRNA expression from 0.52 ± 0.17 to 1.38 ± 0.08 (*p* < 0.001) and from 0.49 ± 0.18 to 1.32 ± 0.07 (*p* < 0.001), but reduced MMP1, MM13, and Cox2 mRNA expression from 0.13 ± 0.01 to 0.04 ± 0.01 (*p* < 0.001), 0.31 ± 0.02 to 0.12 ± 0.02 (*p* < 0.001), and from 0.66 ± 0.03 to 0.13 ± 0.01 (*p* < 0.001), respectively (Figure 5C,D). The findings indicated that the expression trends of Agg, type I/II collagen, and MMP 1/13 were not consistent with that of Cox2, suggesting that DHC partially, rather than completely, relied on JAK1-STAT3-Cox2 to regulate ECM synthesis.

### 2.6. DHC Attenuated Completed ACLT-Induced OA Progression

**Figure 6:**
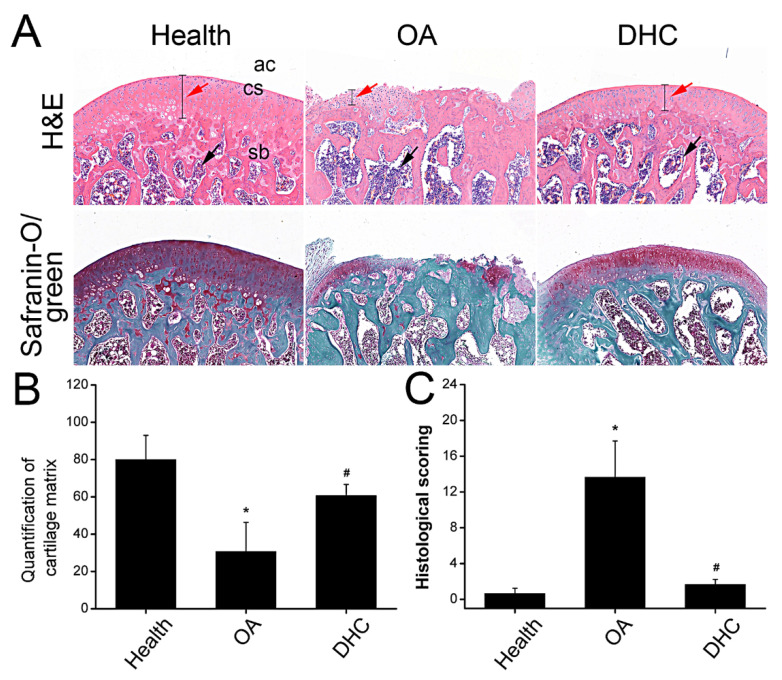
Dehydrocorydaline (DHC) attenuated complete anterior cruciate ligament transection (ACLT)-induced OA progression. (**A**) Cartilage was stained by H&E and safranin-O/fast green (10×). Ac: articular cavity; cs: cartilage surface; sb: subchondral bone. Red arrow: thickness of the cartilage; black arrow: inflammatory cell infiltration. The quantified results of (**B**) cartilage matrix and (**C**) OA histopathology assessment. Data are presented as mean ± SD. *, *p* < 0.05 compared to the healthy group. #, *p* < 0.05 compared to the OA group.

Hematoxylin-eosin (HE) and Safranin-O/fast green staining were performed to carry out the macroscopic analysis. After 6 weeks following complete ACLT surgery, the thickness of the articular cartilage was thinner than the healthy sample, and the cartilage layer was not intact, indicating that most cartilage tissue was degraded (Figure 6A). DHC administration alleviated the progression of OA by retarding the ECM degradation and inflammatory cell infiltration (Figure 6A and Figure S1). Quantification of the cartilage matrix was counted by analyzing Safranin-O/fast green staining. Compared with healthy tissue, quantification of cartilage matrix decreased from 80.03 ± 12.95 to 30.72 ± 15.63 (*p* < 0.05). Nevertheless, DHC administration restored the cartilage matrix to 60.69 ± 6.00 (*p* < 0.05) (Figure 6B). An OA histopathology assessment showed the OA progression scoring. As shown in the Figure 6C, the histological scoring is 13.67 ± 4.04 in the OA group, higher than the healthy group (0.67 ± 0.58) (*p* < 0.05), whereas DHC attenuated the OA progression and decreased the histological scoring to 1.67 ± 0.58 (*p* < 0.05) (Figure 6C), indicating DHC could attenuate the progression of OA.

### 2.7. DHC Inhibited p-JAK1 and p-STAT3 Expression in the Articular Cartilage In Vivo

**Figure 7:**
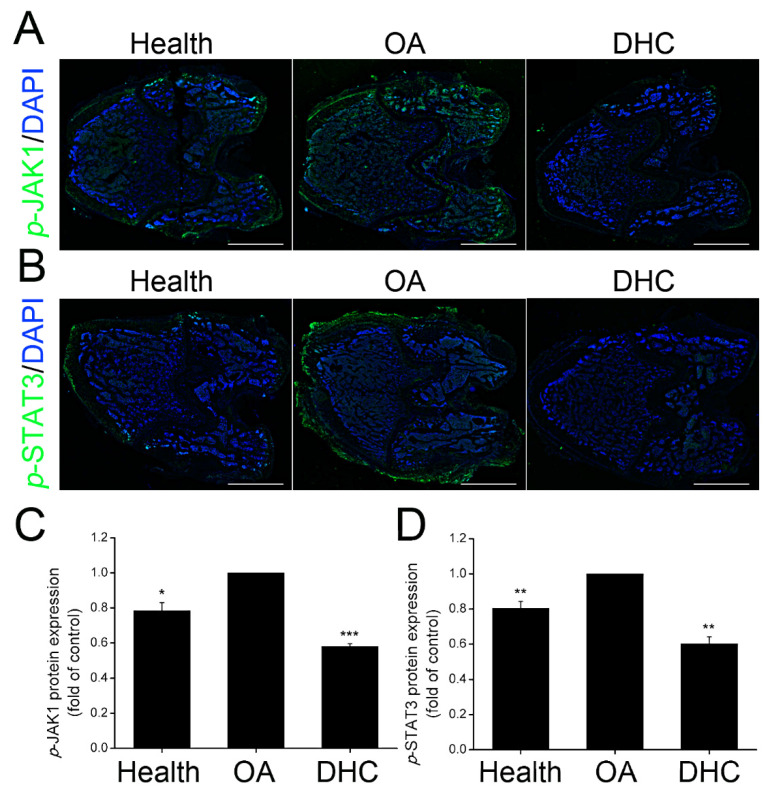
Dehydrocorydaline (DHC) inhibited the *p*-JAK1 and p-STAT3 expression in the complete anterior cruciate ligament transection (ACLT)-induced OA. Protein expression of (**A**) p-JAK1 and (**B**) p-STAT3 detected through immunofluorescence staining. The quantified results of (**C**) p-JAK1 and (**D**) p-STAT3. Scale bar = 2 mm. Data are presented as mean ± SD. *, *p* < 0.05; **, *p* < 0.01; ***, *p* < 0.001 compared to the OA group.

In view of p-JAK1 and p-STAT3 expression in the TNFα-treated human chondrocytes, the protein expression levels were furtherly detected in vivo utilizing immunofluorescence staining in the healthy, OA, and DHC groups (Figure 7A,B). The protein expression of p-JAK1 and p-STAT3 was increased by 27.39% (*p* < 0.05) and 24.22% (*p* < 0.01) in the OA group compared to the healthy group (Figure 7C,D). DHC inhibited *p*-JAK1 and p-STAT3 expression, decreasing them to 58.20 ± 0.01% (*p* < 0.001) and 60.20 ± 0.04% (*p* < 0.01), respectively.

## 3. Discussion

Adequate cartilage matrix content and its structural integrity are the critical factors guaranteeing the normal bearing capacity of articular cartilage. The amount of cell loss induced by cell apoptosis [26] and the limitation of cell proliferation [5], as well as sustained ECM degradation in the cartilage tissue [5], will cause surface discontinuity and even vertical fissures [27] further increase the frictional force on the cartilage surface and aggravate the OA progression. In the current study, DHC’s ability to ameliorate cell loss and ECM degradation is verified in detail. The findings indicated that TNFα treatment significantly reduced cell proliferation and induced the ECM degradation of human chondrocytes in vitro. DHC could improve cell loss through accelerating cell proliferative capacity and ameliorate the ECM degradation by increasing the type II collagen and aggrecan expression and inhibiting the MMP1/13 and Cox2 expression of TNFα-treated human chondrocytes. Simultaneously, RNA sequencing analysis confirmed that DHC weakened the adverse effects of TNFα on the inflammatory factor mRNA expression in the human chondrocytes, e.g., *Il1rn*, *Il13ra2*, *cxcl6/13*. The potential underlying mechanisms were analyzed, and the JAK1-STAT3 signaling pathway was involved in DHC, regulating the abovementioned cell behaviors of TNFα-treated human chondrocytes. In the rat complete ACLT-induced OA models, more inflammatory cells with nuclei stained dark blue and little cytoplasm migrated into the pores in the subchondral bone (SB) and degraded the ECM. DHC administration maintained the structural integrity of the cartilage surface and impaired the OA progression effectively.

The pathology of OA is complex and associated with aging, gender, body weight, and trauma; it is involved in cartilage degeneration, bone remodeling, joint inflammation, and osteophyte formation [28,29]. OA is a common degenerative disease, and older age is one of the risk factors. Moreover, female and/or obese persons are more susceptible to symptomatic knee OA [29]. ACL rupture prior to joint trauma also increases the risk of OA by 12% [29]. All the individuals with OA will experience inflammatory environmental conditions in the knee joint.

Sustained and long-term inflammatory response endanger the regeneration process of cartilage tissue. Inflammatory factors are expressed and secreted into the joint fluid, such as TNFα, IL-1α, IL-6, and IL-1β [30]. The pro-inflammatory factors further up-regulate MMP1, MMP7, MMP8, MMP12, and MMP13 expression and activation, accelerating OA progression and obstructing cartilage regeneration and repair [31]. In the current study, the results also confirmed that the IL-13 receptor, IL-20 receptor, Cox2, MMP3, and MMP13 are highly expressed in the TNFα-treated human chondrocytes (Figure 3D). Surgical implantation is the common clinical treatment manner for serious OA patients. Nevertheless, some clinical data confirm that about 30% of individuals with end-stage OA will suffer long-term limitation of mobility and reduced life quality after undergoing total hip/knee arthroplasty (THA/TKA), which may be due to muscle inflammation susceptibility with highly expressed TNFα and IL-6R [32]. Conversely, the expression of inflammatory factors in the serum of patients undergoing surgical implantation has also been detected by some other researchers. The results indicate that lots of anti-inflammatory factors are highly expressed and secreted into the serum, such as IL-1 receptor antagonist (IL-1RA), IL-2, IL-4, IL-5, IL-6, IL-10, CCL5, CCL11, and vascular endothelial growth factor (VEGF), but no change has been observed in the expression levels of TNFα, interferon-γ, and IL-12, reflecting the rehabilitation process of the cartilage [4]. The researchers hold that the inflammation status of OA patients who undergo joint replacement is controlled, and increased expression levels of IL-6 after the incipient rehabilitation can reflect the anti-inflammatory effect in OA patients [4]. Therefore, the degree of harm to the inflammatory environment post-knee arthroplasty still needs to be explored and discussed.

Inflammatory environmental conditions will activate MMPs to degrade more ECM and accelerate the infiltration of inflammatory cells. Various inflammatory cells migrate to the subchondral bone and even the growth plate [33], secreting more inflammatory factors to induce an adverse feedback loop and aggravate OA progression. The major ECM in the articular cartilage is aggrecan and type II collagen, and the latter can be degraded through MMP3 and MMP13, leading to the structural disruption of the meniscus and cartilage tissue [34]. Impairment of mobility and articular pain caused by OA will greatly reduce individual life quality.

In addition to weakening the inflammatory factor-induced ECM degradation, recruiting sufficient progenitor cells to the articular cartilage is also critical for hindering progressive OA. Mesenchymal stem cells (MSCs) exosomes exhibit excellent immunomodulatory properties and anti-inflammatory abilities and can restore matrix homeostasis to alleviate OA progression [35,36]. Therefore, MSCs are also considered a valuable cell source for OA treatment. In addition, chondrogenic progenitor cells (CPCs) are a unique articular cartilage-derived progenitor cell population with stem cell characteristics and chondrogenic potential [37]. CPCs can be identified by CD29, CD44, CD105, and so on [37]. During the regenerative process, CPCs can migrate to the fracture sites to trigger subsequent cartilage repair. Moreover, the matrix synthesis potential of the CPCs can be further ameliorated through down-regulating the osteogenic transcription factor Runx2 and up-regulating chondrogenic transcription factor Sox9 [37]. Although CPCs exhibit strong potential for cartilage regeneration, the age, gender, and body weight of the individuals will influence its repair capacity [38]. That is, the physical indicators in patients should be taken in account when CPCs are applied in clinical treatment for OA.

Cell base repair attempts in OA are also associated with inflammatory factors, and TNFα or IL-1 blocking may be beneficial for MSCs and CPCs application in OA treatment [38]. Simultaneously, the pathology of OA is manifold and involved in multiple cell behaviors. Therefore, combined therapy with anti-inflammation, growth factors, cell therapy, and special molecules drugs is more suitable for clinical therapy in OA in the future.

## 4. Materials and Methods

### 4.1. Cell Isolation and Culture

Human chondrocytes were supplied by Chongqing University and extracted from four OA patients (age 50~55) undergoing total knee replacement surgery at the First Affiliated Hospital of Chongqing Medical University. Each participant provided informed consent. No donors had inflammatory arthritis or prior knee surgery. The human experiments were performed according to the Ethics Committee of Chongqing University and Huaqiao University. Cartilage tissue was washed with sterile phosphate-buffered saline (PBS) with 5× penicillin and 5× streptomycin sulfate. Subsequently, the tissues were cut into small pieces and digested with 0.06% collagenase type II (BioFroxx, Einhausen, Germany). After 2 h, the chondrocytes were collected and cultured in Dulbecco’s Modified Eagle Medium (DMEM)/F12 (1:1) (Gibco, Carlsbad, CA, USA) supplemented with 10% fetal bovine serum (FBS) (Gibco, Carlsbad, CA, USA). Chondrocytes obtained from different donors were stored separately.

### 4.2. Experimental Design

The chondrocytes were planted in six-well plates (48-well plates for EdU staining) at a cell density of 1 × 10^4^ cells/cm^2^ for 24 h. Then, the medium was replaced with fresh medium with 2% FBS for serum starvation 12 h. Subsequently, the medium was replaced with fresh 2% FBS medium, and 20 ng/mL TNFα (Sigma, Burlington, MA, USA) was added to simulate in vitro OA model. Simultaneously, 10 and 20 μM DHC (dissolved in 0.1% DMSO, Selleck Chemicals, Houston, TX, USA) were applied. Therefore, four groups were established, including the normal group (0.1% DMSO), TNFα group, TNFα + 10 μM DHC group, and TNFα + 20 μM DHC group, and the TNFα group was set as the control group. All the samples were cultured for 48 h, and then EdU staining, RT-qPCR assay, Western blotting, RNA sequencing, and immunocytofluorescence (ICF) staining were performed as described below.

### 4.3. CCK-8 Assay

The effects of DHC on cell viability and the cytotoxicity of TNFα were verified through the Cell Counting Kit-8 (CCK-8) assay (Beyotime, Beijing, China) according to a previous study [39]. Human chondrocytes were planted into the 96-well plates at the cell density of 3000 cells/well and continuously cultured for 24 h. Then, the medium was replaced with 2% FBS medium for serum starvation for 12 h. Subsequently, fresh DMEM/F12 medium with 2% FBS was added, and 1, 5, 10, 20, 50, and 100 μM DHC were applied in the chondrocytes for 48 h to detect cell cytotoxicity. Based on the cytotoxicity assay, 0 (0.1% DMSO), 10 or 20 μM DHC were utilized to treat normal human chondrocytes for 0, 1, 2, 3, and 4 days to analyze cell viability.

### 4.4. EdU Staining and Flow Cytometry

The cell proliferative capacity of human chondrocytes in the normal group, the TNFα group, the TNFα + 10 μM DHC group, and the TNFα + 20 μM DHC group was analyzed through EdU staining and flow cytometry analysis [40,41]. After cell treatment as described above, each well was incubated with EdU staining solution (RiboBio, Guangzhou, China) for 2 h according to the manufacturer’s instructions. Subsequently, cells were fixed using 4% PFA. Later, the medium was replaced by 2 mg/mL glycine and PBS containing 0.5% TritonX-100. Finally, Hoechst 33342 was used to stain the nuclei, and fluorescence images were acquired using fluorescent microscopy (Olympus, Tokyo, Japan). For each group, the percentage of positive cells was counted on five fields by Fiji-Image J analysis software (WS Rasband, National Institute of Health, Bethesda, MD, USA).

For the flow cytometry assay, the human chondrocytes were harvested after experimental treatment and washed with pre-cooled PBS, and then re-suspended in the PBS in a cell density of 1 × 10^6^ cells/mL. Flow cytometry was performed using the cell proliferation assay kit (Keygen Biotech, Jiangsu, China). Subsequently, 1 mL cell suspensions were obtained and centrifuged at 300× *g* × 5 min, and the deposited cells were fixed with 500 μL 70% pre-cooled ethanol at 4 °C for 2 h. Then, cell suspension was centrifuged, and the remaining cells were treated with 100 μL RNase A for 30 min at 37 °C. Finally, 200 μL propidium iodide (PI) was added into the cell suspension, and the samples were incubated at 4 °C for 30 min in darkness. All the samples were analyzed using BD FACS Calibur Flow Cytometer (BD Corp, Franklin Lakes, NJ, USA).

### 4.5. Quantitative Real-Time Polymerase Chain Reaction

The total RNA was extracted from cultured chondrocytes using TRIzol Reagent (Tiangen, Beijing, China). As previously reported [42], 1 μg RNA was reverse-transcribed to complementary DNA (cDNA) by a PrimerScript RT Reagent kit (Takara, Kusatsu, Japan). The mRNA levels of aggrecan, type I/II collagen, MMP1/13, Cox2, JAK1, STAT3, and GAPDH were measured with qRT-PCR with ChamQ Universal SYBR qPCR Master Mix (Vazyme, Shanghai, China) on QuantStadio 6 Pro Real-Time System (Thermo, Waltham, MA, USA). GAPDH was used for normalization. Primers were purchased from Sangon Biotech, and their sequences are listed in Table 1.

### 4.6. Western Blotting Assay

After cell treatment, the cultured chondrocytes were mixed with RIPA buffer (Beyotime, Beijing, China) containing protease/phosphatase inhibitor (Roche, Basel, Switzerland). The concentrations of extracted proteins were measured by BCA Protein Assay Reagent (Beyotime, Beijing, China). Purified proteins (50 μg) were separated in 10% SDS-PAGE gel electophoresis (60 V for stacking gel and 110 V for separation gel) in Mini Protean Tetra Systems (Bio-Rad, Hercules, CA, USA). Next, the separated proteins were transferred to PVDF membranes (Millipore Sigma, Oakville, ON, Canada). After blocking with 5% non-fat milk (Beyotime, Beijing, China), the membranes were incubated with the following primary antibodies at 4 °C overnight: anti-Aggrecan (Abcam, Cambridge, MA, USA, 1:1000), anti-Collagen II (Bioss, Beijing, China, 1:500), anti-MMP13 (Abcam, Cambridge, MA, USA, 1:2000), anti-Cox2 (Abcam, Cambridge, MA, USA, 1:2000), anti-JAK1 (Cell Signaling Technology, Beverly, MA, USA, 1:1000), anti-JAK1 (phospho Tyr1022) (Thermo, Waltham, MA, USA, 1:500), anti-STAT3 (Abcam, Cambridge, MA, USA, 1:1000), anti-STAT3 (phospho Y705) (Abcam, Cambridge, MA, USA, 1:1000), and anti-GAPDH (Sangon Biotech, Shanghai, China, 1:500). Finally, the membranes were incubated with horseradish peroxidase (HRP)-conjugated secondary antibodies (Bioss, Beijing, China, 1:5000) and visualized with the ChemiDoc XRS system (Bio-Rad, Hercules, CA, USA).

### 4.7. RNA Sequencing

RNA samples were extracted in the normal group, the TNFα group, and the TNFα + 10 μM DHC group and utilized for RNA sequencing to verify the changes in mRNA expression profiles. RNA sequencing was carried out in BGI Co.; LTD (Wuhan, China) utilizing BGISEQ-500. Subsequently, the sequencing data was analyzed using an R language program. Differently expressed genes between the normal and TNFα groups, or TNFα and TNFα + 10 μM DHC groups, were screened. Furthermore, GO function enrichment analysis and KEGG signal pathway enrichment analysis were performed utilizing Dr. Tom and Omicshare online software.

### 4.8. Immunocytofluorescence Staining

Protein expression levels of *p*-JAK1 and p-STAT3 in the normal group, the TNFα group, and the TNFα + 10 μM DHC group were detected with ICF staining according to our previous study [3]. The samples were incubated with the primary antibodies anti-STAT3 (phospho Y705) (Abcam, Cambridge, MA, USA, 1:300) and anti-JAK1 (phospho Tyr1022) (Cell Signaling Technology, Beverly, MA, USA, 1:50) overnight at 4  °C. Then, the cells were incubated with FITC-conjugated secondary antibody (Beyotime, Shanghai, China, 1:300) for 1 h at room temperature (RT). Subsequently, cell nuclei were visualized with DAPI (Beyotime, Shanghai, China) staining for 15 min, and fluorescence images were obtained using fluorescent microscopy (Olympus, Tokyo, Japan). For each group, the fluorescence intensity was counted on five fields by Fiji-Image J analysis software (WS Rasband, National Institute of Health, Bethesda, MD, USA).

### 4.9. Inhibition of JAK1-STAT3 Signaling Pathway

The chondrocytes were planted in six-well plates at a cell density of 1 × 10^4^ cells/cm^2^ for 24 h. After serum starvation for 12 h, the medium was replaced with fresh DMEM/F12 with 2% FBS. Subsequently, 20 ng/mL TNFα (Sigma, Burlington, MA, USA) with/without 20 μM S3I-201 (S3I, inhibitor of JAK1-STAT3 pathway, Selleck Chemicals, Houston, TX, USA) was added to the medium. Simultaneously, 10 μM DHC (Selleck Chemicals, Houston, TX, USA) with/without 20 μM S3I-201 (S3I, inhibitor of JAK1-STAT3 pathway, Selleck Chemicals, Houston, TX, USA) were applied. Cell responses in normal chondrocytes are shown in Figure 2. Therefore, four groups were established, including the TNFα group, the TNFα + 10 μM DHC group, the TNFα + 20 μM S3I group, and the TNFα + 10 μM DHC + 20 μM S3I group. The cell proliferation and mRNA expression levels of aggrecan, type I/II collagen, MMP1/13, and Cox2 in the human chondrocytes were detected using EdU staining and RT-qPCR analysis, respectively.

### 4.10. Rat Complete ACL Transection Model

All the animal experiments were performed following the Ethics Committee of Chongqing University and Huaqiao University. A total of 12 rats were randomly divided into three groups: a healthy group, OA group, and DHC group. The Sprague Dawley (SD) rat OA models were established through complete ACL transection surgery in the OA and DHC groups according to our previous study [13]. After ACLT surgery, 50 μL 1 mM DHC (dissolved in sterile saline solution) was injected into the knee articular cavity from the third to sixth week, whereas an equal volume of saline solution was used in the OA group and the healthy group. Drug administration was performed daily in the third week, and then once every 3 days in the next 3 weeks. Subsequently, articular cartilage tissues were harvested after DHC administration for 6 weeks, and micro-evaluation was carried out.

### 4.11. Hematoxylin-Eosin and Masson Staining

Articular cartilage was analyzed 6 weeks after ACLT surgery. Cartilage was fixed with 4% paraformaldehyde (PFA) for 48 h, dehydration with 30% sucrose (Sangon Biotech, Shanghai, China) for 48 h, and embedded by Tissue-Tek OCT compound (Sakura Finetechnical, Tokyo, Japan). The cartilage specimens were cut to a thickness of 10 μm by a freezing microtome (Leica, Wetzlar, Germany). Then, the freezing sections were conducted with hematoxylin and eosin (H&E) staining (Solarbio, Beijing, China) and Safranin O/Fast Green staining (Solarbio, Beijing, China) according to the manufacturer’s instructions. Histological scoring was performed according to a previous study [27].

### 4.12. Immunohistofluorescence Staining

Protein expression levels of p-JAK1 and p-STAT3 in vivo were further detected through immunohistofluorescence (IHF) staining according to a previous study [43]. The freezing sections were subjected to heat-induced antigen retrieval using citrate buffer (Solarbio, Beijing, China) and incubated with 5% bovine serum albumin (BSA) for 1 h at 37 °C. Next, sections were incubated with primary antibodies specific for anti-STAT3 (phospho Y705) (Abcam, Cambridge, MA, USA, 1:300) and anti-JAK1 (phospho Tyr1022) (Cell Signaling Technology, Beverly, MA, USA, 1:50) overnight at 4  °C. After washing, sections were incubated with FITC-conjugated secondary antibody (Beyotime, Shanghai, China, 1:300). Cell nuclei were visualized by DAPI (Beyotime, Shanghai, China) staining, and fluorescent images were obtained using fluorescent microscopy (Olympus, Tokyo, Japan). For each group, the fluorescence intensity was counted on five fields by Fiji-Image J analysis software (WS Rasband, National Institute of Health, Bethesda, MD, USA).

### 4.13. Statistical Analysis

The data were tested using OriginPro 8.0 software and represented in the manuscript as the mean ± standard deviation (SD). Statistical analysis was performed by one-way ANOVA analysis of variance. Moreover, *p* < 0.05 was set as the critical significance level. All the experiments were repeated at least three times.

## 5. Conclusions

In summary, DHC can effectively weaken the adverse effects induced by TNFα on cell proliferation and ECM synthesis in human chondrocytes. Moreover, DHC significantly increased aggrecan and type II collagen expression, but inhibited type I collagen, MMP1, MMP13, Cox2, and other inflammatory factor expression levels in TNFα-treated human chondrocytes. The JAK1-STAT3 signaling pathway was confirmed to be the underlying mechanism involved in DHC, regulating cell proliferation and ECM synthesis of the TNFα-treated human chondrocytes. In addition, DHC also exhibited positive effects on ECM synthesis in ACLT-induced OA models and expression levels of *p*-JAK1 and p-STAT3 in vivo. This study supplied a potential treatment strategy for OA in a clinic.

## Figures and Tables

**Table 1:** List of primer sequences used for quantitative real-time PCR.

Gene.	Primer Sequence	Amplicon Length
Aggrecan	F: 5′- ACTCTGGGTTTTCGTGACTCT -3′	81 bp
	R: 5′- ACACTCAGCGAGTTGTCATGG -3′	
Collagen 1	F: 5′- CTGGAAGAGTGGAGAGTACTG -3′	143 bp
	R: 5′- TGCTGATGTACCAGTTCTTCTG -3′	
Collagen 2	F: 5′- CCAGATGACCTTCCTACGCC -3′	186 bp
	R: 5′- TTCAGGGCAGTGTACGTGAAC -3′	
MMP1	F: 5′- AAAATTACACGCCAGATTTGCC -3′	82 bp
	R: 5′- GGTGTGACATTACTCCAGAGTTG -3′	
MMP13	F: 5′- CCAGACTTCACGATGGCATTG -3′	137 bp
	R: 5′- GGCATCTCCTCCATAATTTGGC -3′	
Cox2	F: 5′- ATGCTGACTATGGCTACAAAAGC -3′	90 bp
	R: 5′- TCGGGCAATCATCAGGCAC -3′	
Jak1	F: 5′- CCACTACCGGATGAGGTTCTA -3′	213 bp
	R: 5′- GGGTCTCGAATAGGAGCCAG -3′	
STAT3	F: 5′- ACCAGCAGTATAGCCGCTTC -3′	124 bp
	R: 5′- GCCACAATCCGGGCAATCT -3′	
GAPDH	F: 5′- GGATTTGGTCGTATTGGG -3′	218 bp
	R: 5′- GCTCCTGGAAGATGGTGAT -3′	

## Data Availability

All data are included in the article.

## Supplementary Materials

The following supporting information can be downloaded at: https://www.mdpi.com/article/10.3390/ijms23137268/s1.
