# Supplementary material for: Dehydrocorydaline Accelerates Cell Proliferation and Extracellular Matrix Synthesis of TNFα-Treated Human Chondrocytes by Targeting Cox2 through JAK1-STAT3 Signaling Pathway
Source: Int J Mol Sci. 2022 Jun 30;23(13):7268. doi: 10.3390/ijms23137268 (PMC9267121; doi:10.3390/ijms23137268)
Supplement: Supplementary file 1 [file ijms-23-07268-s001.zip › ijms-1785480-supplementary.pdf]

Supplementary figures and legends

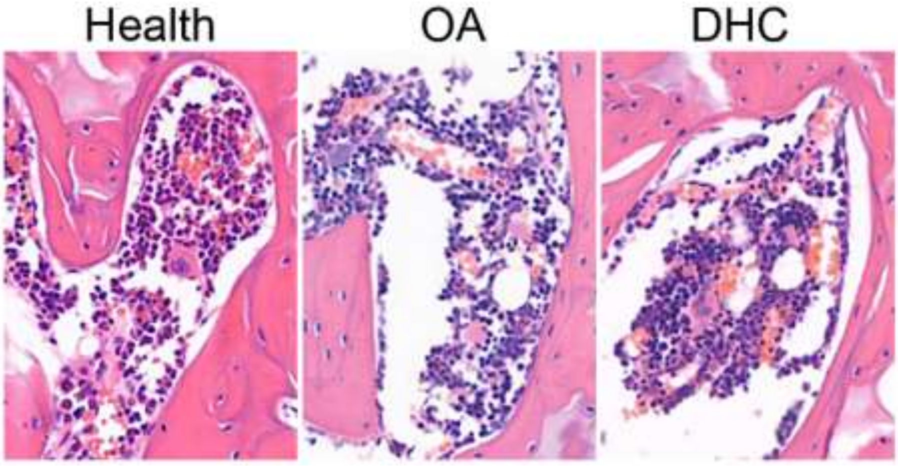

Figure S1. Dehydrocorydaline (DHC) attenuated complete anterior cruciate ligament transection (ACLT)-induced OA progression. More dark blue punctate inflammatory cells exited in the OA tissue, whereas DHC administration inhibited cell invasion of inflammatory cells into the pores of subchondral bone.
